# Age-adjusted high-dose chemotherapy followed by autologous stem cell transplantation or conventional chemotherapy with R-MP as first-line treatment in elderly primary CNS lymphoma patients – the randomized phase III PRIMA-CNS trial

**DOI:** 10.1186/s12885-023-11193-7

**Published:** 2023-08-18

**Authors:** Lisa K Isbell, Roswitha Uibeleisen, Alexander Friedl, Elvira Burger, Tatja Dopatka, Florian Scherer, Andras Orban, Eliza Lauer, Natalie Malenica, Inna Semenova, Annika Vreden, Elke Valk, Julia Wendler, Simone Neumaier, Heidi Fricker, Abed Al Hadi El Rabih, Cora Gloggengießer, Daniela Hilbig, Sabine Bleul, Joachim Weis, Dennis Gmehlin, Matthias Backenstrass, Sebastian Wirtz, Gabriele Ihorst, Jürgen Finke, Gerald Illerhaus, Elisabeth Schorb

**Affiliations:** 1 Department Medicine I, Faculty of Medicine, Medical Center-University of Freiburg, University of Freiburg, Hugstetter Straße 55, 79106 Freiburg, Germany; 2 Clinic of Hematology, Oncology and Palliative Care, Klinikum Stuttgart, Kriegsbergstraße 60, 70174 Stuttgart, Germany; 3 Department of Endocrinology, Diabetology and Geriatrics, Klinikum Stuttgart, Prießnitzweg 24, 70374 Bad Cannstatt, Germany; 4 Stuttgart Cancer Center - Tumorzentrum Eva Mayer-Stihl, Klinikum Stuttgart, Kriegsbergstraße 60, 70174 Stuttgart, Germany; 5 Endowed Professorship Self-Help Research, Comprehensive Cancer Center, Faculty of Medicine and Medical Center, University of Freiburg, Freiburg, Germany; 6 Institute for Clinical Psychology, Klinikum Stuttgart, Prießnitzweg 24, 70374 Bad Cannstatt, Germany; 7 Clinical Trials Unit, Faculty of Medicine and Medical Center, University of Freiburg, Elsässer Straße 2, 79110 Freiburg, Germany

**Keywords:** Primary central nervous system lymphoma (PCNSL), High-dose chemotherapy (HCT), Autologous stem cell transplantation (ASCT), Elderly patients, Transplant eligibility

## Abstract

**Background:**

Older primary central nervous system lymphoma (PCNSL) patients have an inferior prognosis compared to younger patients because available evidence on best treatment is scarce and treatment delivery is challenging due to comorbidities and reduced performance status. High-dose chemotherapy and autologous stem cell transplantation (HCT-ASCT) after high-dose methotrexate (MTX)-based immuno-chemotherapy has become an increasingly used treatment approach in eligible elderly PCNSL patients with promising feasibility and efficacy, but has not been compared with conventional chemotherapy approaches. In addition, eligibility for HCT-ASCT in elderly PCNSL is not well defined. Geriatric assessment (GA) may be helpful in selecting patients for the best individual treatment choice, but no standardized GA exists to date. A randomized controlled trial, incorporating a GA and comparing age-adapted HCT-ASCT treatment with conventional chemotherapy is needed.

**Methods:**

This open-label, multicenter, randomized phase III trial with two parallel arms will recruit 310 patients with newly diagnosed PCNSL > 65 years of age in 40 centers in Germany and Austria. The primary objective is to demonstrate that intensified chemotherapy followed by consolidating HCT-ASCT is superior to conventional chemotherapy with rituximab, MTX, procarbazine (R-MP) followed by maintenance with procarbazine in terms of progression free survival (PFS). Secondary endpoints include overall survival (OS), event free survival (EFS), (neuro-)toxicity and quality of life (QoL). GA will be conducted at specific time points during the course of the study. All patients will be treated with a pre-phase rituximab-MTX (R-MTX) cycle followed by re-assessment of transplant eligibility. Patients judged transplant eligible will be randomized (1:1). Patients in arm A will be treated with 3 cycles of R-MP followed by maintenance therapy with procarbazine for 6 months. Patients in arm B will be treated with 2 cycles of MARTA (R-MTX/AraC) followed by busulfan- and thiotepa-based HCT-ASCT.

**Discussion:**

The best treatment strategy for elderly PCNSL patients remains unknown. Treatments range from palliative to curative but more toxic therapies, and there is no standardized measure to select patients for the right treatment. This randomized controlled trial will create evidence for the best treatment strategy with the focus on developing a standardized GA to help define eligibility for an intensive treatment approach.

**Trial registration:**

German clinical trials registry DRKS00024085 registered March 29, 2023.

**Supplementary Information:**

The online version contains supplementary material available at 10.1186/s12885-023-11193-7.

## Background

Median age at diagnosis is above 60 years in primary diffuse large B-cell lymphoma (DLBCL) of the central nervous system (PCNSL), a rare lymphoma confined to the cerebral parenchyma, leptomeninges, eyes or spinal cord [1, 2]. Standard treatment consists of high-dose methotrexate- (MTX) based immunochemotherapy followed by consolidation treatment [3–7]. So far, there is no clear standard of care due to reduced evidence in that age group and treatment decisions are often challenging because of comorbidities and poor performance status (PS) due to PCNSL diagnosis [2]. Induction treatment with the MATRix combination (MTX, cytarabine (AraC), thiotepa (TT), and rituximab) followed by consolidation treatment with high-dose chemotherapy and autologous stem cell transplantation (HCT-ASCT) significantly improved outcomes in patients aged 70 years or younger in the randomized IELSG32 trial of the International Extranodal Lymphoma Study Group (IELSG) [8, 9]. However, for patients > 65 years, especially if presenting with comorbidities and poor performance status, this regimen is often considered too toxic. The PRIMAIN phase II, single arm study created some evidence on conventional therapy with R-MP and procarbazine maintenance with 1- and 2-year progression free survival (PFS) rates of 46.3% and 37.3% and respective overall survival (OS) rates after 1 and 2 years of 56.7% and 47% [10]. Efforts have been made to conduct trials specifically designed for elderly PCNSL patients with the focus on developing age-adapted treatment regimens with curative potential. Prospective evidence on age-adjusted HCT-ASCT consolidation revealed feasibility and efficacy in selected elderly patients in a pilot study and subsequent multicenter phase II MARTA study with 1-year PFS and OS rates in the intention to treat population of 58.8% and 62.7% respectively and with a toxicity profile that was comparable to other treatment protocols [10–14].

However, toxicities - mainly infectious complications which commonly occur in the 1st treatment cycle - remain major challenges in the treatment of PCNSL patients. In an international retrospective analysis investigating the MATRix regimen in routine clinical practice the 1st cycle of MATRix was associated with the most severe toxicities, with 6% of patients requiring admission to the intensive care unit in comparison to only 1 admission during cycle 2–4 of MATRix [5]. In the MARTA study 15/51 (29.4%) patients did not reach consolidation treatment mostly due to serious adverse events, mainly comprising infectious and neurovascular events[13]. The incidence of early infectious toxicities during PCNSL induction treatment is likely to be associated with impairment of PS and neurocognitive disorder (due to PCNSL). Furthermore, the immunosuppressive effects of corticosteroid exposure, frequently prescribed at the time of initial diagnosis of PCNSL might contribute to this effect. Thus, to reduce the frequently observed toxicity in the 1st cycle of chemotherapy and achieve disease stabilization as well as improvement of clinical PS a pre-phase treatment is implemented in this trial. A pre-phase treatment was implemented in 3 randomized controlled trials, 1 in PCNSL patients, with the goal to improve the PS and treatment delivery [15–17].

Defining fitness for transplant in elderly PCNSL patients is very challenging because established assessment tools are missing. Geriatric assessments (GA) covering functional status, comorbidity, cognitive function, psychological state, social support and nutritional status have been recently incorporated into clinical trials for elderly systemic DLBCL patients [18–21]. Two comorbidity scores, the Charlson Comorbidity Index (CCI [22]) and the Cumulative Illness Rating Scale-Geriatric (CIRS-G [23]) as well as one geriatric screening tool, the Geriatric 8 (G8 [24]), have only been assessed retrospectively in PCNSL patients, identifying only the CIRS-G ≥ 8 to be associated with decreased OS and PFS [25]. Our study group retrospectively investigated the influence on treatment outcome of ECOG PS, Lachs geriatric screening [26] as well as Barthel Index of Activity of Daily Living (ADL) [27] in the prospective MARTA and MARiTA studies. A composite sum score of **E**COG PS (1 point if ECOG > 1), **B**arthel Index of ADL (1 point if less than full points in ADL) and **L**achs geriatric screening (1 point if Lachs > 3), named the **EBL** Score > 1, seems to be associated with a higher chance of premature end of treatment (EOT) [28]. Nevertheless, to our knowledge, an extensive GA has never been carried out in a PCNSL study and will be implemented in the PRIMA-CNS trial. In addition, we will test the neurologic assessment in Neuro-oncology (NANO) scale for its utility to supplement the International PCNSL Collaborative Group’s (IPCG) response criteria for magnetic resonance imaging (MRI) evaluation of response [29] as well as premorbid performance status and premorbid functional status assessed with Instrumental Activity of Daily Living (IADL) [30, 31]. The difficult question of whether a patient is fit enough to tolerate HCT-ASCT can be discussed in a study board with a geriatrician and oncology experts.

Randomized controlled trials are urgently needed to improve therapy options in this subgroup of elderly patients and define eligibility for age-adapted intensive treatment approaches [32]. We therefore propose this randomized, multicenter, phase III trial, comparing age-adjusted HCT-ASCT with the conventional R-MP protocol comprising procarbazine maintenance as first-line treatment in elderly PCNSL patients.

## Methods

### Study design

This is an open-label, multicenter, randomized phase III trial with two parallel arms. The study design was approved by the leading ethics committee (Ethik-Kommission Albert-Ludwigs-Universität Freiburg, Germany) and the local ethics committees of the participating centers. The protocol was also subject to authorization by the competent authorities as mandatory by federal law. All participants have to provide written informed consent. The trial was assigned the EudraCT number 2020-001181-10 and is registered at German clinical trials registry (DRKS00024085, registration date March 29th, 2023).

### Study objectives and endpoints

The primary objective of this trial is to demonstrate superiority (in terms of PFS) of a shorter but more intensive treatment regimen comprising 2 cycles of MARTA (R-MTX/AraC) followed by busulfan and thiotepa-based HCT-ASCT, compared to standard treatment with R-MP and procarbazine maintenance. The primary endpoint PFS is defined as time from randomization to disease progression or death of any cause, with censoring at the last date the patient was seen alive and free of disease progression.

Secondary efficacy endpoints are OS, event free survival (EFS), defined as time from randomization to premature EOT due to any reason, lymphoma progression or death, whichever occurs first, censoring at the last date the patient was seen event-free, remission rate (complete remission (CR) and partial remission (PR)) after 2 cycles of R-MP (arm A) / 2 cycles of MARTA prior to consolidation treatment (arm B) (measured at response assessment (RA) I) and after 3 cycles of R-MP (arm A) / after HCT-ASCT (arm B) (measured at RA II). Tumor response will be assessed by gadolinium-enhanced brain MRI according to the IPCG response criteria [33] and will be evaluated by central independent radiological review, not involved in the conception of the study. Secondary safety endpoints include (serious) adverse events, toxicity (according to National Cancer Institute’s Common Terminology Criteria for Adverse Events (NCI-CTCAE) v5.0), and QoL (measured by EORTC QLQ-C30 [34] and -BN20 [35]) as well as rate of unplanned hospital admissions and length of hospital stays. Additionally, neurocognitive impairment in general will be measured by Montreal Cognitive Assessment (MoCA [36]) and will be supplemented by the Trail Making Test A and B [37], the Rey-Osterrieth-Complex-Figure-Test [38] and a verbal fluency test [38] in order to assess psychomotor speed, executive functions and visuoconstruction in more detail.

### Geriatric assessment

We will focus especially on establishing GA tools to help define transplant eligibility. Therefore a GA covering functional status (ADL, IADL), comorbidity (CIRS-G, CCI, HCT-CI [39]), cognitive function (MoCA), psychological state (depression PHQ9 [40], anxiety GAD7 [41]), social support (SSUK [42]), nutritional status (weight, height, weight loss questionnaire [43]) and polypharmacy (Lachs geriatric screening) domains will be checked at specific timepoints during the study.

### Patient involvement

In a pre-study phase patient’s priorities and information preferences were assessed to optimize the patient trial information in cooperation with the Department of Self-help Research of the Comprehensive Cancer Center Freiburg (CCCF). In addition, patients’ representatives of a national patient advocacy organisation (German Leukemia and Lymphoma Aid DLH) were involved to support us in improving the informed consent material of the trial. As a result, a checklist for the informed consent discussion with the patient was developed to support the study physicians. At regular intervals patients and their families will be informed about the process of the trial in patient information events. Our positive experience with the participation of patients in the pre-study phase encouraged us to involve patients’ representatives (German Leukemia and Lymphoma Aid, DLH) throughout the whole period of the clinical trial in the data monitoring committee (DMC).

### Eligibility criteria

Immunocompetent patients with newly-diagnosed primary DLBCL of the central nervous system, age > 70 years or age 65–70 years if not eligible for more intensive treatment (e.g. OptiMATe trial [17]) with an ECOG PS ≤ 2 are eligible. An ECOG PS > 2 due to PCNSL symptoms is also acceptable for inclusion. For further details on inclusion and exclusion criteria please see Table 1.

A special focus of the trial lies on developing tools to help define transplant eligibility, therefore additional randomization criteria were implemented after the pre-phase treatment:

1. Patients eligible for HCT-ASCT defined by the EBL score (at most one of the 3 following conditions may apply: ECOG PS > 1, Barthel Index of ADL < 20 and Lachs geriatric screening > 3), improvement of PS after pre-phase treatment or clinical judgement by the treating physician after discussion with the study expert team in a study board. Of note, for the Barthel Index of ADL the version with a maximum of 20 points will be used.
2. No evidence of disease progression after pre-phase treatment.

**Table 1:** Inclusion and exclusion criteria

Inclusion criteria	1. Immunocompetent patients with newly-diagnosed primary DLBCL of the central nervous system. 2. Age > 70 years or age 65–70 years if not eligible for more intensive treatment (e.g. OptiMATe trial). 3. Histologically or cytologically assessed diagnosis of B-cell lymphoma by local pathologist. 4. Diagnostic sample obtained by stereotactic or surgical biopsy, CSF cytology examination or vitrectomy. 5. Disease exclusively located in the CNS. 6. At least 1 measurable lesion. 7. ECOG-Performance Status ≤ 2 (ECOG PS > 2 due to PCNSL symptoms is acceptable) 8. Patients possibly eligible for HCT-ASCT as judged by the treating physician. 9. Written informed consent obtained according to international guidelines and local laws by patient or authorized legal representative in case patient is temporarily legally not competent due to his or her disease.
Exclusion criteria	1. Congenital or acquired immunodeficiency including HIV infection and previous organ transplantation. 2. Systemic lymphoma manifestation (outside the CNS). 3. Primary vitreoretinal lymphoma or primary leptomeningeal lymphoma without manifestation in the brain parenchyma or spinal cord. 4. Previous or concurrent malignancies with the exception of surgically cured carcinoma in situ or other kinds of cancer without evidence of disease for at least 5 years. 5. Previous systemic Non-Hodgkin lymphoma at any time. 6. Inadequate renal function (creatinine clearance < 60 ml/min). 7. Inadequate bone marrow, cardiac, pulmonary or hepatic function according to investigator´s decision. 8. Active hepatitis B or C disease. 9. Concurrent treatment with other experimental drugs or participation in an interventional clinical trial with administration of study medication within the last thirty days before the start of this study. 10. Third space fluid accumulation > 500 ml. 11. Hypersensitivity to study treatment or any component of the formulation. 12. Taking any medications likely to cause interactions with the study medication. 13. Known or persistent abuse of medication, drugs or alcohol. 14. Active COVID-19-infection or non-compliance with the prevailing hygiene measures regarding the COVID-19 pandemic. 15. Patients without legal capacity and who are unable to understand the nature, significance and consequences of the study and without designated legal representative. 16. Previous participation in this trial. 17. Persons who are in a relationship of dependency/employment to the sponsor and/ or investigator. 18. Any familial, sociological or geographical condition potentially hampering compliance with the study protocol and follow-up schedule. 19. Fertile patients refusing to use safe contraceptive methods during the study.

### Randomization methodology

Randomization will be performed after the pre-phase treatment, stratified by ECOG performance score (ECOG 0/1 vs. ECOG ≥ 2), in blocks with randomly varying block sizes in a ratio of 1:1. The block lengths will be documented separately and will not be disclosed to the centers. Stratification by study center will not be performed due to a large number of centers with presumably few patients. The randomization lists will be provided by the trial biometrician and uploaded into secuTrial® by the data management. The randomization sequence will be produced by validated programs based on the Statistical Analysis System (SAS®).

A randomization form will be created in secuTrial®. Performance of randomization will be documented electronically at the study site in secuTrial® to guarantee concealment of the next treatment allocation. Randomization will take place after the pre-phase treatment once the additional randomization criteria are verified.

### Treatment schedule

**Fig. 1:**
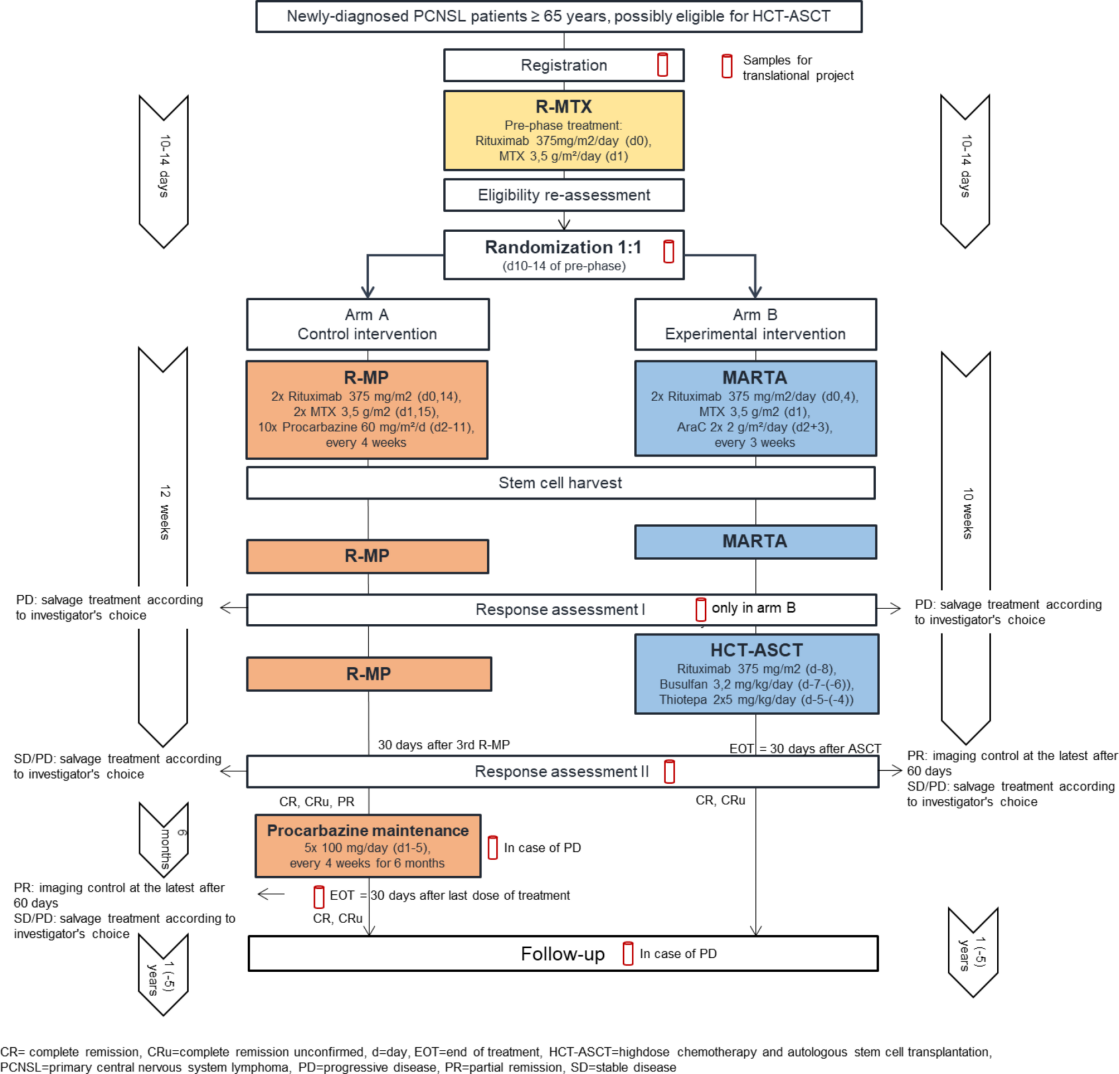
Intervention Scheme

Figure 1 shows the intervention scheme. Patients will either receive the standard treatment (Arm A) comprising 3 cycles of R-MP followed by procarbazine maintenance or the experimental treatment (Arm B) comprising 2 cycles of MARTA followed by HCT-ASCT consolidation. Treatment duration is depicted on the left (arm A) and right side (arm B). The red test tube indicates time points when samples for the translational project are to be collected (for details see translational research program).

### Treatment plan

All enrolled patients will receive 1 cycle of pre-phase treatment with rituximab 375 mg/m^2^ and MTX 3.5 g/m^2^ to achieve disease stabilization and improvement of clinical PS.

#### Arm A (control intervention)

Patients in the control arm (arm A) will receive 3 cycles of R-MP (rituximab 375 mg/m² i.v. **d0**,**14**; MTX 3,5 g/m² i.v. **d1,15**; procarbazine 60 mg/m²/d p.o. **d2-11**) every 4 weeks followed by maintenance treatment with procarbazine 100 mg absolute/d p.o. **d1-5** every 4 weeks for 6 cycles (duration of maintenance treatment 6 months). Stem cell harvest will be performed after cycle 1 of R-MP according to local standard procedure. In case of unsuccessful stem cell harvest after cycle 1, further attempts can be made after cycle 2 and 3.

#### Arm B (experimental intervention)

Patients in the experimental arm will receive 2 cycles of R-MTX/AraC (rituximab 375 mg/m² i.v. d0, 4; MTX 3,5 g/m² i.v. d1, AraC 2 × 2 g/m²/d i.v. d2 + 3) every 3 weeks followed by consolidating HCT-ASCT with rituximab 375 mg/m^2^ d-8, busulfan 3.2 mg/kg/d i.v. d-7 and d-6 and thiotepa 5 mg/kg/d i.v. d-5 and d-4.

Stem cell harvest will be performed after cycle 1 of MARTA according to local standard procedure. In case of unsuccessful stem cell harvest after cycle 1, stem cell harvest can be performed after cycle 2.

### Assessments and follow-up

The following parameters will be gathered at each visit: ECOG PS, vital signs, thorough physical and neurological examination including the NANO Scale, laboratory tests and adverse events. At screening, we will assess also premorbid PS as well as premorbid functional status. Before randomization, patients will be re-evaluated for HCT-ASCT eligibility.

Imaging evaluation by whole brain gadolinum-enhanced MRI will be conducted after cycle 2 of R-MP (arm A) / cycle 2 of MARTA (arm B) as well as after cycle 3 of R-MP (arm A) / 30 days after ASCT (arm B).

In addition to these timepoints, patients with clinical signs of progression at any time during the study treatment or if study treatment is delayed for more than 2 weeks during induction treatment will receive subsequent gadolinium-enhanced brain MRI to confirm radiologic disease status.

During follow-up gadolinium-enhanced brain MRI will be performed every 3 months in year 1–2, every 6 months in year 3–5 and annually thereafter. Tumor size and location(s) will be assessed only at screening and in case of progressive disease (PD)). Tumor number (singular/multiple) will only be assessed at screening.

Independent pathologic and radiologic review are implemented in this trial to assure high-level methodical and quality standards.

Further information on assessments during the trial and the follow-up period are given in the appendix (see supplementary material, additional file 1: trial flow chart arm A and arm B).

### Translational research program

Within this trial, a comprehensive translational research program is implemented to improve our understanding of the pathogenetic factors that drive lymphomagenesis and to establish innovative biomarkers that improve risk stratification and outcome prediction. We will comprehensively explore tumor biopsies to characterize molecular subgroups of patients with certain (epi-)genetic alterations. Additionally, we will apply targeted next-generation sequencing to circulating tumor DNA (ctDNA) obtained from blood plasma and cerebrospinal fluid (CSF) throughout the treatment course to investigate its role as prognostic biomarker (Fig. 1) [44].

### Sample size calculation

The sample size calculation is based on the primary endpoint PFS. The control intervention is the R-MP protocol, as previously reported [10]. Within the PRIMAIN study, the proportion of patients being free of lymphoma relapse/progression or death 12 months after diagnosis was about 50%. Assuming improved toxicity profile and protocol adherence by the addition of the pre-phase treatment and a greater familiarity with the protocol, we expect a higher PFS rate at 12 months of 55% for the control arm in the trial proposed herein. Results of the MARTA study report a PFS rate of about 60% at 12 months after diagnosis. Meeting the above mentioned effect of the pre-phase treatment, we conservatively expect a PFS rate above 65% (67.5% for calculation) 12 months after randomization, corresponding to hazard ratio of experimental versus control of 0.66. Considering an exponential survival time distribution, a two-sided alpha = 0.05 and power = 0.8 (recruitment time 60 months, follow-up (FU) time from randomization between 12 and 72 months, up to 12 months after the randomization of the last patient), 178 events on total need to be observed, and 118 patients per arm need to be randomized in a 1:1 ratio (total n = 236) and need to have complete FU. To account for possible drop outs (e.g. loss of FU or violation of inclusion/exclusion criteria), we aim to randomize 260 patients. Patients lost to FU will be included in the full analysis set (FAS), but may lead to a slightly reduced treatment effect. Assumptions of the dropout rate and of the rate of registered patients not reaching randomization are also based on the results of our previous studies. The latter is expected to range around 15%, thus we plan to include 310 patients in the trial pre-phase. The program used for the calculation is nquery 7.0, the test method applied is the log-rank two group test of equal exponential survival.

### Statistical analysis

The primary analysis will be performed according to the intention-to-treat (ITT) principle in the full analysis set (FAS). The FAS includes all randomized patients in whom therapy after randomization was started. Patients will be analysed in the treatment arms to which they were randomized, irrespective of whether they refused or discontinued the treatment or whether other protocol deviations are known.

The analysis of the primary endpoint PFS will be conducted in the FAS according to the intention-to-treat principle. The treatment effect will be estimated and tested by Cox regression. The regression model will include treatment and, for adjustment, the stratification variable ECOG (0/1 vs. ≥ 2) as independent variables. As estimate of the effect size, the hazard ratio (arm A vs. arm B) will be calculated with a corresponding asymptotic two-sided 95% confidence interval (CI). The two-sided test on the difference between the experimental arm A and the control arm B at two-sided significance level of 5% will be based on the corresponding asymptotic two-sided 95% CI from the Cox regression model.

Additionally, PFS rates in the two treatment arms will be estimated by the Kaplan-Meier method.

The primary analysis is conducted irrespective of the occurrence of intercurrent events, consistent with the treatment policy strategy of the estimands framework described in the ICH E9 (R1) addendum. Intercurrent events comprise early termination of treatment due to toxicity, treatment failure or patient´s wish. These events are captured in the secondary endpoint EFS in accordance with the composite strategy.

As a sensitivity analysis, the analysis will be repeated in the per-protocol (PP) set, excluding patients with major protocol violations.

Secondary endpoints will be analyzed descriptively. EFS and OS will be analyzed in the same way as described for PFS. Remission after 2 cycles of induction treatment will be determined after the 2nd cycle of R-MP in arm A and after the 2nd cycle of MARTA in arm B (= RA I in both arms). The endpoint remission after completion of the 3rd cycle of R-MP (arm A) / consolidating HCT-ASCT (arm B) will be determined within 25–35 days after the start of the 3rd cycle of R-MP (arm A) / on day 30 after ASCT (arm B).

Sensitivity analyses will be performed by additionally including other relevant prognostic variables: gender, LDH level (≤ vs. > above upper normal limit), involvement of deep structures of the brain (yes vs. no) and protein count of cerebrospinal fluid (≤ vs. > above upper normal limit), to adjust for potential confounding in the regression models proposed above for the primary and the secondary endpoints.

Safety analyses will be performed in the safety population (SAF). Patients in the SAF are analyzed as belonging to the treatment arm defined by treatment received. Patients are included in the respective treatment arm, if treatment was started.

### Quality assurance and safety

The data management will be done with secuTrial® (https://www.secutrial.com/). During data entry, the data will be checked by so-called edit checks. There are study-specific reports programmed and generated with secuTrial® or SAS software that allow to check for completeness, consistency, plausibility, as well as protocol violations and other distinctive problems (e.g. cumulative missing data). All programs which can be used to influence the data or data quality will be validated.

### Data monitoring committee

An independent Data Monitoring Committee (DMC) has been established, that consists of two medical scientists and one statistician with longstanding experience in clinical trials. The DMC’s function is to monitor the study’s course and if necessary, make recommendations to the coordinating or principal investigator for study continuation, modification or discontinuation. The DMC members will be informed about patient recruitment, adherence to the protocol, observed serious adverse events and deaths by receiving the development safety update reports (DSURs) at regular intervals.

## Discussion

Treatment strategies for elderly PCNSL patients > 65 years are not well defined reflecting in inferior outcome in comparison to younger patients. Treatment of this vulnerable patient population is challenging because of a frequently poor PS and comorbidities [31]. A recent scoping review summarized the current study pool available, evaluating different types and combinations of chemotherapy in elderly PCNSL patients. In total, 6 randomized controlled trials (RCT) with 1.346 patients, 26 prospective (with 1.366 patients) and 24 retrospective studies (with 2.629 patients) were identified. Of these, only 6 studies (1 completed and 1 ongoing RCT (with 447 patients), 1 completed and 1 ongoing prospective single arm study (with 65 patients), and 2 retrospective single arm studies (with 122 patients) evaluated HCT-ASCT. The current study pool is, however, not conclusive in terms of the most effective treatment options for elderly patients. Main limitations were (very) small sample sizes and heterogeneous patient populations in terms of age ranges (particularly in RCTs) limiting the applicability of the results to the target population (elderly PCNSL patients) [32]. To date, there is only 1 randomized trial specifically designed for elderly patients with PCNSL [14]: Omuro et al. tested 2 MTX-based regimens of different intensity in 98 PCNSL patients ≥ 60 years, however, neither treatment regimen comprises HCT-ASCT. To date, only 1 completed prospective pilot study [11], 1 recently completed prospective phase II study [12, 13] and 2 retrospective studies [45, 46] specifically investigated intensified consolidation treatment with HCT-ASCT in elderly PCNSL patients. Retrospective data of an international multicenter analysis investigating HCT-ASCT in eligible elderly PCNSL patients report 2-year OS and PFS rates of 86.5% and 62.0%, respectively [45]. Similar outcomes were observed in a retrospective study from the UK which included 31% of patients over the age of 65 years [46]. Results of the recently completed phase II MARTA study, examining an age-adapted HCT-ASCT approach in the elderly PCNSL population in a multicenter study, proved this approach feasible and effective with PFS and OS rates compared to that of younger cohorts [13, 47]. Nevertheless, toxicity with infectious as well as neurovascular complications was observed and resulted in premature end of treatment prior to consolidation in nearly 30% of patients.

Judgment of who can profit from an intensive therapy approach remains difficult because standardized tools to define transplant eligibility are missing. We found GA scores like CIRS-G, ECOG PS, Barthel Index of ADL and Lachs geriatric screening to be associated with premature end of treatment as well as decreased PFS and OS in the MARTA and MARiTA study population, whereas age did not seem to play a major role[28]. We have included a pre-phase treatment with rituximab and MTX in the current study for all patients with the aim to improve PS and reduce treatment-related toxicities. Pre-phase treatments have been performed in other trials for elderly systemic DLBCL patients with successful reduction of toxicity [15, 17, 48]. After the pre-phase treatment patients will be reassessed for transplant eligibility, with the help of the above mentioned GA and study board. Geriatric assessment tools have been incorporated in clinical trials mainly for elderly systemic DLBCL patients with association to survival outcomes. There is emerging evidence for tailoring chemotherapy intensity according to GAs. Recently the Italian Lymphoma Foundation developed a tool to assess frailty that incorporates age, comorbidities, ADL, IADL and categorizes patients as “fit”, “unfit”, or “frail”. When comparing outcome parameters in a cohort of 1163 elderly systemic DLBCL patients, frail patients showed 3 year OS rates of 43% in comparison to 75% of fit patients. However, treatment regimen in that study were diverse [19, 20]. To validate these findings GA scores will be implemented in an upcoming SWOG 1918 trial comparing Acazidicine + R-miniCHOP vs. R-miniCHOP in elderly systemic DLBCL patients [21]. Regarding PCNSL, Farhi et al. found that the CIRS-G with a cut-off of ≥ 8 was associated with decreased OS and PFS in a cohort of 35 elderly PCNSL patients that were treated with a least 1 dose of MTX [25]. We will assess 3 comorbidity scales (CCI, CIRS-G as well as transplant specific HCT-CI) within this proposed trial to create more evidence for the role of comorbidity scores on treatment outcome alone and in combination with other GA scores.

We aim to demonstrate the superiority of the MARTA induction protocol followed by consolidation with HCT-ASCT compared to the established standard therapy with conventional chemotherapy with R-MP and procarbazine maintenance, in terms of PFS. The implemented GA will create evidence on how to define transplant eligibility. This is the first randomized controlled trial investigating HCT-ASCT in this population of elderly patients. Results of this multicenter randomized trial will either change the standard of care to an intensive and shorter treatment approach or redefine R-MP as a proven treatment standard.

### Electronic supplementary material

Below is the link to the electronic supplementary material.

Additional file 1: Flow Chart - control intervention arm A (table S1) and experimental intervention arm B (table S2)

## Data Availability

As this is a trial protocol report data sharing is not applicable to this article as no datasets were analyzed during the current study.

## Abbreviations

ADL: Activities of Daily Living
ASCT: Autologous stem cell transplantation
CCI: Charlson Comorbidity Index
CHOP: cyclophosphamide, doxorubicine, vincristine, prednisone
CI: Confidence interval
CIRS-G: Cumulative Illness Rating Scale-Geriatric
CNS: Central Nervous System
CR: Complete remission
ctDNA: Circulating Tumor Deoxyribonucleic Acid
d: Day
DMC: Data Monitoring Committee
DLBCL: Diffuse large B-cell lymphoma
ECOG: Eastern Cooperative Oncology Group
EFS: Event Free Survival
EORTC QLQ-C30: European Organization for Research and Treatment of Cancer Questionnaire Core-30
EORTC QLQ-BN20: European Organization for Research and Treatment of Cancer Questionnaire Brain Tumor module 20
FAS: Full Analysis Set FU: follow-up
GA: Geriatric assessment
AraC: cytarabine
MTX: methotrexate
H(D)CT: High-dose chemotherapy
HCT-CI: Hematopoietic Cell Transplantation-Comorbidity Index
HIV: Human immunodeficiency virus
IELSG: International Extranodal Lymphoma Study Group
IPCG: International PCNSL Collaborative Group
ITT: Intention to treat
MATRix: MTX, AraC, Thiotepa, Rituximab
MoCA: Montreal Cognitive Assessment
MRI: Magnetic Resonance Imaging
NANO: Neurologic Assessment in Neuro-Oncology
NCI-CTCAE: National Cancer Institute - Common toxicity criteria for adverse events
OS: Overall survival
PCNSL: Primary central nervous system lymphoma
PD: Progressive disease
PFS: Progression free survival
PP: Per-protocol
PR: Partial remission
PS: Performance status
QoL: Quality of life
RA: Response Assessment
RCT: Randomized Controlled Trial
SAS: Statistical Analysis System
SD: Stable disease
TMT-A/-B: Trail Making Test A/B

## References

[1] Mendez JS, Ostrom QT, Gittleman H, Kruchko C, DeAngelis LM, Barnholtz-Sloan JS (2018). The elderly left behind-changes in survival trends of primary central nervous system lymphoma over the past 4 decades. Neuro Oncol.

[2] Houillier C, Soussain C, Ghesquieres H, Soubeyran P, Chinot O, Taillandier L (2020). Management and outcome of primary CNS lymphoma in the modern era: an LOC network study. Neurology.

[3] Roth P, Martus P, Kiewe P, Mohle R, Klasen H, Rauch M (2012). Outcome of elderly patients with primary CNS lymphoma in the G-PCNSL-SG-1 trial. Neurology.

[4] Siegal T, Bairey O (2019). Primary CNS lymphoma in the Elderly: the challenge. Acta Haematol.

[5] Schorb E, Fox CP, Kasenda B, Linton K, Martinez-Calle N, Calimeri T et al. Induction therapy with the MATRix regimen in patients with newly diagnosed primary diffuse large B-cell lymphoma of the central nervous system - an international study of feasibility and efficacy in routine clinical practice. Br J Haematol. 2020;published online ahead of print, 2020 Jan 29. 10.1111/bjh.16451 31997308

[6] Roth P, Hoang-Xuan K (2014). Challenges in the treatment of elderly patients with primary central nervous system lymphoma. Curr Opin Neurol.

[7] Olivier G, Clavert A, Lacotte-Thierry L, Gardembas M, Escoffre-Barbe M, Brion A (2014). A phase 1 dose escalation study of idarubicin combined with methotrexate, vindesine, and prednisolone for untreated elderly patients with primary central nervous system lymphoma. The GOELAMS LCP 99 trial. Am J Hematol.

[8] Ferreri AJ, Cwynarski K, Pulczynski E, Ponzoni M, Deckert M, Politi LS (2016). Chemoimmunotherapy with methotrexate, cytarabine, thiotepa, and rituximab (MATRix regimen) in patients with primary CNS lymphoma: results of the first randomisation of the International Extranodal Lymphoma Study Group-32 (IELSG32) phase 2 trial. Lancet Haematol.

[9] Ferreri AJM, Cwynarski K, Pulczynski E, Fox CP, Schorb E, La Rosee P (2017). Whole-brain radiotherapy or autologous stem-cell transplantation as consolidation strategies after high-dose methotrexate-based chemoimmunotherapy in patients with primary CNS lymphoma: results of the second randomisation of the International Extranodal Lymphoma Study Group-32 phase 2 trial. Lancet Haematol.

[10] Fritsch K, Kasenda B, Schorb E, Hau P, Bloehdorn J, Mohle R (2017). High-dose methotrexate-based immuno-chemotherapy for elderly primary CNS lymphoma patients (PRIMAIN study). Leukemia.

[11] Schorb E, Kasenda B, Ihorst G, Scherer F, Wendler J, Isbell L (2020). High-dose chemotherapy and autologous stem cell transplant in elderly patients with primary CNS lymphoma: a pilot study. Blood Adv.

[12] Schorb E, Finke J, Ihorst G, Kasenda B, Fricker H, Illerhaus G (2019). Age-adjusted high-dose chemotherapy and autologous stem cell transplant in elderly and fit primary CNS lymphoma patients. BMC Cancer.

[13] Schorb E, et al. High-dose chemotherapy and autologous stem cell transplantation in Elderly and Fit Primary CNS lymphoma patients - a Multicenter Study by the Cooperative PCNSL Study Group (MARTA study). American Society of Hematology (ASH) abstract; 2022.

[14] Omuro A, Chinot O, Taillandier L, Ghesquieres H, Soussain C, Delwail V (2015). Methotrexate and temozolomide versus methotrexate, procarbazine, vincristine, and cytarabine for primary CNS lymphoma in an elderly population: an intergroup ANOCEF-GOELAMS randomised phase 2 trial. Lancet Haematol.

[15] Peyrade F, Bologna S, Delwail V, Emile JF, Pascal L, Ferme C (2017). Combination of ofatumumab and reduced-dose CHOP for diffuse large B-cell lymphomas in patients aged 80 years or older: an open-label, multicentre, single-arm, phase 2 trial from the LYSA group. Lancet Haematol.

[16] Pfreundschuh M, Trumper L, Kloess M, Schmits R, Feller AC, Rube C (2004). Two-weekly or 3-weekly CHOP chemotherapy with or without etoposide for the treatment of elderly patients with aggressive lymphomas: results of the NHL-B2 trial of the DSHNHL. Blood.

[17] Wendler J, Fox CP, Valk E, Steinheber C, Fricker H, Isbell LK (2022). Optimizing MATRix as remission induction in PCNSL: de-escalated induction treatment in newly diagnosed primary CNS lymphoma. BMC Cancer.

[18] Hurria A, Cirrincione CT, Muss HB, Kornblith AB, Barry W, Artz AS (2011). Implementing a geriatric assessment in cooperative group clinical cancer trials: CALGB 360401. J Clin Oncol.

[19] Tucci A, Martelli M, Rigacci L, Riccomagno P, Cabras MG, Salvi F (2015). Comprehensive geriatric assessment is an essential tool to support treatment decisions in elderly patients with diffuse large B-cell lymphoma: a prospective multicenter evaluation in 173 patients by the Lymphoma Italian Foundation (FIL). Leuk Lymphoma.

[20] Merli F, Luminari S, Tucci A, Arcari A, Rigacci L, Hawkes E (2021). Simplified geriatric Assessment in older patients with diffuse large B-Cell lymphoma: the prospective Elderly Project of the Fondazione Italiana Linfomi. J Clin Oncol.

[21] Brem EA, Li H, Beaven AW, Caimi PF, Cerchietti L, Alizadeh AA (2022). SWOG 1918: a phase II/III randomized study of R-miniCHOP with or without oral azacitidine (CC-486) in participants age 75 years or older with newly diagnosed aggressive non-hodgkin lymphomas - aiming to improve therapy, outcomes, and validate a prospective frailty tool. J Geriatr Oncol.

[22] Charlson ME, Pompei P, Ales KL, MacKenzie CR (1987). A new method of classifying prognostic comorbidity in longitudinal studies: development and validation. J Chronic Dis.

[23] Linn BS, Linn MW, Gurel L (1968). Cumulative illness rating scale. J Am Geriatr Soc.

[24] Bellera CA, Rainfray M, Mathoulin-Pelissier S, Mertens C, Delva F, Fonck M (2012). Screening older cancer patients: first evaluation of the G-8 geriatric screening tool. Ann Oncol.

[25] Farhi J, Laribi K, Orvain C, Hamel JF, Mercier M, Del Sutra A (2018). Impact of front line relative dose intensity for methotrexate and comorbidities in immunocompetent elderly patients with primary central nervous system lymphoma. Ann Hematol.

[26] Lachs MS, Feinstein AR, Cooney LM, Drickamer MA, Marottoli RA, Pannill FC (1990). A simple procedure for general screening for functional disability in elderly patients. Ann Intern Med.

[27] Mahoney FI, Barthel DW (1965). Functional evaluation: the Barthel Index. Md State Med J.

[28] Vreden A, I L, Ihorst G, Kerkhoff A, Fricker H, Malenica N, Finke J, Illerhaus G, Schorb E. Deutsche, Österreichische und Schweizerische Gesellschaften für Hämatologie und Medizinische Onkologie. Oncol Res Treat 2021;44(I–III).

[29] Nayak L, DeAngelis LM, Brandes AA, Peereboom DM, Galanis E, Lin NU (2017). The neurologic Assessment in Neuro-Oncology (NANO) scale: a tool to assess neurologic function for integration into the Response Assessment in Neuro-Oncology (RANO) criteria. Neuro Oncol.

[30] Lawton MP, Brody EM (1969). Assessment of older people: self-maintaining and instrumental activities of daily living. Gerontologist.

[31] Martinez-Calle N, Isbell LK, Cwynarski K, Schorb E (2022). Advances in treatment of elderly primary central nervous system lymphoma. Br J Haematol.

[32] Schorb E, Isbell LK, Illerhaus G, Ihorst G, Meerpohl JJ, Grummich K (2021). Treatment regimens for Immunocompetent Elderly patients with primary Central Nervous System Lymphoma: a scoping review. Cancers (Basel).

[33] Abrey LE, Batchelor TT, Ferreri AJ, Gospodarowicz M, Pulczynski EJ, Zucca E et al. Report of an international workshop to standardize baseline evaluation and response criteria for primary CNS lymphoma. J Clin Oncol. 2005;23(22):5034-43. 10.1200/JCO.2005.13.524 15955902

[34] Aaronson NK, Ahmedzai S, Bergman B, Bullinger M, Cull A, Duez NJ (1993). The European Organization for Research and Treatment of Cancer QLQ-C30: a quality-of-life instrument for use in international clinical trials in oncology. J Natl Cancer Inst.

[35] Taphoorn MJ, Claassens L, Aaronson NK, Coens C, Mauer M, Osoba D (2010). An international validation study of the EORTC brain cancer module (EORTC QLQ-BN20) for assessing health-related quality of life and symptoms in brain cancer patients. Eur J Cancer.

[36] Nasreddine ZS, Phillips NA, Bedirian V, Charbonneau S, Whitehead V, Collin I (2005). The Montreal Cognitive Assessment, MoCA: a brief screening tool for mild cognitive impairment. J Am Geriatr Soc.

[37] Correa DD, Maron L, Harder H, Klein M, Armstrong CL, Calabrese P (2007). Cognitive functions in primary central nervous system lymphoma: literature review and assessment guidelines. Ann Oncol.

[38] Strauss E, Sherman EM, Spreen O. A compendium of neuropsychological tests: Administration, norms, and commentary. American chemical society; 2006.

[39] Berro M, Arbelbide JA, Rivas MM, Basquiera AL, Ferini G, Vitriu A (2017). Hematopoietic cell transplantation-specific Comorbidity Index predicts morbidity and mortality in autologous stem cell transplantation. Biol Blood Marrow Transplant.

[40] Lowe B, Kroenke K, Herzog W, Grafe K (2004). Measuring depression outcome with a brief self-report instrument: sensitivity to change of the Patient Health Questionnaire (PHQ-9). J Affect Disord.

[41] Spitzer RL, Kroenke K, Williams JB, Lowe B (2006). A brief measure for assessing generalized anxiety disorder: the GAD-7. Arch Intern Med.

[42] Philipp R, Mehnert A, Lehmann C, Oechsle K, Bokemeyer C, Krull A (2016). Detrimental social interactions predict loss of dignity among patients with cancer. Support Care Cancer.

[43] Fried LP, Tangen CM, Walston J, Newman AB, Hirsch C, Gottdiener J (2001). Frailty in older adults: evidence for a phenotype. J Gerontol A Biol Sci Med Sci.

[44] Mutter JA, Alig SK, Esfahani MS, Lauer EM, Mitschke J, Kurtz DM et al. Circulating tumor DNA profiling for detection, risk stratification, and classification of Brain Lymphomas. J Clin Oncol. 2022:JCO2200826. 10.1200/JCO.22.00826 PMC10419411 36542815

[45] Schorb E, Fox CP, Fritsch K, Isbell L, Neubauer A, Tzalavras A (2017). High-dose thiotepa-based chemotherapy with autologous stem cell support in elderly patients with primary central nervous system lymphoma: a european retrospective study. Bone Marrow Transplant.

[46] Kassam S, Chernucha E, O’Neill A, Hemmaway C, Cummins T, Montoto S (2017). High-dose chemotherapy and autologous stem cell transplantation for primary central nervous system lymphoma: a multi-centre retrospective analysis from the United Kingdom. Bone Marrow Transplant.

[47] Illerhaus G, Ferreri AJM, Binder M, Borchmann P, Hasenkamp J, Stilgenbauer S (2022). Effects on Survival of Non-Myeloablative Chemoimmunotherapy compared to high-dose chemotherapy followed by autologous stem cell transplantation (HDC-ASCT) as consolidation therapy in patients with primary CNS lymphoma - results of an International Randomized Phase III Trial (MATRix/IELSG43). Blood.

[48] Lakshmaiah KC, Asati V, Babu KG, Jacob DL (2018). Role of prephase treatment prior to definitive chemotherapy in patients with diffuse large B-cell lymphoma. Eur J Haematol.

