# Supplementary material for: Age-adjusted high-dose chemotherapy followed by autologous stem cell transplantation or conventional chemotherapy with R-MP as first-line treatment in elderly primary CNS lymphoma patients – the randomized phase III PRIMA-CNS trial
Source: BMC Cancer. 2023 Aug 18;23:767. doi: 10.1186/s12885-023-11193-7 (PMC10436648; doi:10.1186/s12885-023-11193-7)
Supplement: Supplementary file 1 — Additional file 1: Flow Chart - control intervention arm A (table S1) and experimental intervention arm B (table S2) [file 12885_2023_11193_MOESM1_ESM.pdf]

Additional Table S1 Visit schedule and assessments – Flowchart: Control Treatment - Arm A

**PERIODS**		**SCREENING**	**TREATMENT – ARM A including MAINTENANCE**								**EOT**		**FOLLOW-UP**	
	Duration	14 days	10-14 days		28 days	28 days		28 days		6 months			at least 1 (max. 6) year(s) after RA II	
**Visits**	**Title in eCRF**	**Screening**	**Pre-phase**	**Rando-mization**	**Visit 1[1]**	**Visit 2[1]**	**RA I[1]**	**Visit 3[1]**	**RA II[1]**	**Maintenance Visits**	**EOT Visit[1]**	**Pre-mat-ure EOT[1]**	**FU Yr 1-2**	**FU from Yr3[2]**
**Time window for assessments / Interval of treatment administration**	Time / Section of CTP	d-14 until d0	d0-1	d10-14	d0-15[3] of cycle 1	d0-15[3] of cycle 2	d25-27 of cycle 2	d0-15[3] of cycle 3	d25-27 of cycle 3	Visits every 3 months/ Treatment d1-5 every 4 weeks	d30 after last administration of IMP		every 3 mo (± 14 d)	every 6 mo (± 30 d)
**Informed consent[5]**	15.3	x												
**Inclusion/exclusion criteria**	4.2, 4.3,	x		x										
**Registration**	5.2.1	x												
**Treatment administration**	6.1		x		x	x		x	x[6]	x[6]				
**Demographics, Medical History**	7.8.1 7.8.2	x												
**Physical and neurological examination*/****	7.8.3	x	x		x	x		x	x	x	x	x	x	x
**Vital signs***	7.8.4	x	x		x	x		x	x	x	x	x	x	x
**Body height and weight**	7.8.4	x	x		x	x		x	x	x				
**Performance status (Karnofsky and ECOG)**	7.8.5	x			x	x		x	x	x	x	x	x	x
**Premorbid performance status (Karnofsky and ECOG)**	7.8.5	x												
**Lachs geriatric screening**	7.8.6	x		x										
**Barthel Index of ADL**	7.8.6	x		x					x	x	x	x	x[13]	x[13]
**IADL (premorbid + current status)**	7.8.6	x												
**Weight loss questionnaire**	7.8.6	x												
**CIRS-G**	7.8.6	x												
**Charlson Comorbidity Index (CCI)**	7.8.6	x												
**HCT-ASCT eligibility assessment[26]**	7.8.14			x										
**Hematology[7]/Clinical chemistry[8]***	7.8.15	x	x	x	x	x		x	x	x	x	x	x	x
**Creatinine, estimated GFR (MDRD)[8]**	7.8.15	x	x		x	x		x						
**Hepatitis B/C and HIV serology***	7.8.15	x												

PERIODS		SCREENING	TREATMENT – ARM A including MAINTENANCE								EOT		FOLLOW-UP	
	Duration	14 days	10-14 days		28 days	28 days		28 days		6 months			at least 1 (max. 6) year(s) after RA II	
**Visits**	**Title in eCRF**	**Screening**	**Pre-phase**	**Rando-mization**	**Visit 1[1]**	**Visit 2[1]**	**RA I[1]**	**Visit 3[1]**	**RA II[1]**	**Maintenance Visits**	**EOT Visit[1]**	**Pre-mat-ure EOT[1]**	**FU Yr 1-2**	**FU from Yr3[2]**
**Time window for assessments / Interval of treatment administration**	Time / Section of CTP	d-14 until d0	d0-1	d10-14	d0-15[3] of cycle 1	d0-15[3] of cycle 2	d25-27 of cycle 2	d0-15[3] of cycle 3	d25-27 of cycle 3	Visits every 3 months/ Treatment d1-5 every 4 weeks	d30 after last administration of IMP		every 3 mo (± 14 d)	every 6 mo (± 30 d)
**Electrocardiogram (ECG)***	7.8.16	**x**												
**Whole body plethysmography[24]**	7.8.16	**x**												
**Echocardiography***	7.8.16	**x**												
**Testicular ultrasound***	7.8.17	**x**												
**Abdominal ultrasound[9]***	7.8.18		**x**		**x**	**x**		**x**						
**Whole brain MRI and response statement according to IPCG (local and central radiology)**	7.8.19	**x**					**x**		**x**	**x[14]**	**x**	**x[15]**	**x[16]**	**x[16]**
**Imaging (CT neck to pelvis)[10]***	7.8.19	**x**												
**Shipment to central pathology[25]**	7.8.20	**x**												
**Bone marrow examination[27]***	7.8.21	**x**												
**Slit lamp examination[11]**	7.8.22	**x**					**x[11]**		**x[11]**		**x[11]**	**x[11]**		
**CSF examination[12]**	7.8.23	**x**		**(x)[22]**			**x[11]**		**x[11]**		**x[11]**	**x[11]**		
**MoCA[13]**	7.8.8.1	**x**							**x**			**x**	**x[13]**	**x[13]**
**PHQ9[13], GAD7[13], SSUK[13]**		**x**							**x**			**x**	**x[13]**	**x[13]**
**QLQ (EORTC QLQ-C30 BN20)[13]**	7.8.24	**x**							**x**			**x**	**x[13]**	**x[13]**
**Neuropsychological battery[17]**	7.8.8.2	**x**							**x**			**(x)[17]**	**(x)[13]**	**(x)[13]**
**NANO**		**x**		**x**				**x**	**x**			**x**	**x[13]**	**x[13]**
**Subjective evaluation of trial participation**	7.8.9	**x[18]**							**x[19]**		**x[19]**	**x[19]**		
**Stem cell harvest**	7.8.24				**x[20]**									
**HCT-CI**	7.8.6.2	**x**												
**Translational program[21]**	7.9	**x[21]**		**x[21]**					**x[21]**	**(x)[21]**	**x[21]**	**(x)[21]**	**(x)[21]**	**(x)[21]**
**Concomitant medication**	6.3	**x**	**x**											
**Adverse events (CTCAE v. 5.0)**	10	**x**	**x**										**x[23]**	

ADL=Activities of daily living; AE=Adverse Event; ASCT=Autologous stem cell transplantation; CIRS-G = Cumulative Illness Rating Scale Geriatric; CSF=Cerebrospinal fluid; CT=Computed Tomography; CTCAE v.5.0=Common Terminology Criteria for Adverse Events version 5.0; d=Day(s); EORTC QLQ-C30=Quality of Live Questionnaire; EOT=End of Treatment; FU=Follow-up; GAD7=General Anxiety Disorder 7; GFR=Glomerular Filtration Rate; HCT-ASCT=High-dose chemotherapy and autologous stem-cell transplantation; HCT-CI=Hematopoietic Cell Transplantation- Comorbidity Index; HIV=Human immunodeficiency virus; IADL= Instrumental Activities of Daily Living; IMP=Investigational Medicinal Product; IPCG=International PCNSL Collaborative Group; LDH=Lactate dehydrogenase; MDRD=Modification of Diet in Renal Disease; mo=Month(s); MoCA=Montreal Cognitive Assessment; MRI=Magnetic Resonance Imaging; NANO= Neurologic Assessment in Neuro-Oncology; PHQ9=Personal Health Questionnaire form 9; QLQ-BN 20=brain tumor module Questionnaire; QOQ=Quality of Life Questionnaire; SSUK=Social Support in Chronic Illness Questionnaire; yr=Year; RA=Response Assessment; For additional details see corresponding numbering (footnotes).

Additional Table S2 Visit schedule and assessments – Flowchart: Experimental Treatment - Arm B

**PERIODS**		**Screening**	**TREATMENT – ARM B**						**EOT**		**FOLLOW-UP**	
	Duration	14 days	10-14 days		21 days	21 days		39 days			at least 1 (max. 6) year(s) after RA II	
**Visits**	**Title in eCRF**	**Screening**	**Pre-phase**	**Rando-mization**	**Visit 1[1]**	**Visit 2[1]**	**RA I[1]**	**Visit 3[1]**	**RA II = EOT Visit[1]**	**premature EOT Visit[1]**	**FU Yr 1-2**	**FU from Yr3[2]**
**Time window for assessments / Interval of treatment administration**	Time / Section	d-14 until day 0	d0-1	d10-14	d0-4[3] of cycle 1	d0-4[3] of cycle 2	d18-20 of cycle 2	d-8 to d0 of HCT-ASCT[4]	d30 after ASCT	d30 after last adminis-tration of IMP	every 3 mo (± 14 d)	every 6 mo (± 30 d)
**Informed consent[5]**	15.3	x										
**Inclusion/exclusion criteria**	4.2, 4.3,	x		x								
**Registration**	7.3.4	x										
**Treatment administration**	6.1		x		x	x		x				
**Demographics, Medical History**	7.8.1, 7.8.2	x										
**Physical and neurological examination***	7.8.3	x	x		x	x		x	x	x	x	x
**Vital signs***	7.8.4	x	x		x	x		x	x	x	x	x
**Body height and weight**	7.8.4	x	x		x	x		x				
**Performance status (Karnofsky and ECOG)**	7.8.5	x			x	x		x	x	x	x	x
**Premorbid performance status (Karnofsky and ECOG)**	7.8.5	x										
**Lachs geriatric screening**	7.8.6	x		x								
**Barthel Index of ADL**	7.8.6	x		x					x	x	x[13]	x[13]
**IADL (premorbid + current status)**	7.8.6	x										
**Weight loss questionnaire**	7.8.6	x										
**CIRS-G**	7.8.6	x										
**Charlson Comorbidity Index (CCI)**	7.8.6	x										
**HCT-ASCT eligibility assessment[26]**	7.8.14			x								
**Hematology[7]/Clinical chemistry[8]***	7.8.15	x	x	x	x	x		x	x	x	x	x
**Creatinine, estimated GFR (MDRD)[8]**	7.8.15	x	x		x	x		x				
**Hepatitis B/C and HIV serology***	7.8.15	x										
**Electrocardiogram (ECG)***	7.8.16	x						x				

PERIODS		Screening	TREATMENT – ARM B						EOT		FOLLOW-UP	
	Duration	14 days	10-14 days		21 days	21 days		39 days			at least 1 (max. 6) year(s) after RA II	
**Visits**	**Title in eCRF**	**Screening**	**Pre-phase**	**Rando-mization**	**Visit 1**[1]	**Visit 2**[1]	**RA I**[1]	**Visit 3**[1]	**RA II = EOT Visit**[1]	**premature EOT Visit**[1]	**FU Yr 1-2**	**FU from Yr3**[2]
**Time window for assessments / Interval of treatment administration**	Time / Section	d-14 until day 0	d0-1	d10-14	d0-4[3] of cycle 1	d0-4[3] of cycle 2	d18-20 of cycle 2	d-8 to d0 of HCT-ASCT[4]	d30 after ASCT	d30 after last adminis-tration of IMP	every 3 mo (± 14 d)	every 6 mo (± 30 d)
**Whole body plethysmography**[24]*	7.8.16	x						x				
**Echocardiography***	7.8.16	x						x				
**Testicular ultrasound***	7.8.17	x										
**Abdominal ultrasound**[9]*	7.8.18		x		x	x						
**Whole brain MRI and response statement according to IPCG (local and central radiology)**	7.8.19	x					x		x	x[15]	x[16]	x[16]
**Imaging (CT neck to pelvis)**[10]*	7.8.19	x										
**Shipment to central pathology**[25]	7.8.20	x										
**Bone marrow examination**[27]*	7.8.21	x										
**Slit lamp examination**[11]	7.8.22	x					x[11]		x[11]	x[11]		
**CSF examination**[12]	7.8.23	x		(x)[22]			x[11]		x[11]	x[11]		
**MoCA**[13]	7.8.8.1	x							x	x	x[13]	x[13]
**PHQ9**[13]**, GAD7**[13]**, SSUK**[13]		x							x	x	x[13]	x[13]
**QLQ (EORTC QLQ-C30 + BN20)**[13]	7.8.7	x							x	x	x[13]	x[13]
**Neuropsychological battery**[17]	7.8.8.2	x							x	x	(x)[13]	(x)[13]
**NANO**		x		x				x	x	x	x[13]	x[13]
**Subjective evaluation of trial participation**	7.8.9	x[18]							x[19]	x[19]	x[19]	
**Stem cell harvest**	7.8.24				x[20]							
**HCT-CI**	7.8.6.2	x						x				
**Translational program**[21]	7.9	x[21]		x[21]			x[21]		x[21]	(x)[21]	(x)[21]	(x)[21]
**Concomitant medication**	6.3	x	x									
**Adverse events (CTCAE V. 5.0)**	10	x	x								x[23]	

ADL=Activities of daily living; AE=Adverse Event; ASCT=Autologous stem cell transplantation; CIRS-G = Cumulative Illness Rating Scale Geriatric; CSF=Cerebrospinal fluid; CT=Computed Tomography; CTCAE v. 5.0=Common Terminology Criteria for Adverse Events version 5.0; d=Day(s); EORTC QLQ-C30=Quality of Live Questionnaire; EOT=End of Treatment; FU=Follow-up; GAD7=General Anxiety Disorder 7; GFR=Glomerular Filtration Rate; HCT-ASCT=High-dose chemotherapy and autologous stem-cell transplantation; HCT-CI=Hematopoietic Cell Transplantation- Comorbidity Index; HIV=Human immunodeficiency virus; IADL=Instrumental Activities of Daily Living; IMP=Investigational Medicinal Product; IPCG=International PCNSL Collaborative Group; LDH=Lactate dehydrogenase; MDRD=Modification of Diet in Renal Disease; mo=Month(s); MoCA=Montreal Cognitive Assessment; MRI=Magnetic Resonance Imaging; NANO=Neurologic Assessment in Neuro-Oncology; PHQ9=Personal Health

Questionnaire form 9; QLQ-BN 20=brain tumor module Questionnaire; QOQ=Quality of Life Questionnaire; SSUK=Social Support in Chronic Illness Questionnaire; yr=Year; RA=Response Assessment; For additional details see corresponding numbering (footnotes).

* not to be documented in the eCRF

** Physical examination is recommended to be performed according to the flow chart; detailed findings concerning these examinations must only be documented in the eCRF at screening. At other visits, in case of clinically relevant abnormal findings, the investigator has to document an AE on the AE-page in the eCRF

[1] Deviations ± 5 days are allowed

[2] Also after termination of study, follow up every 3 months (year 2), every 6 months (year 3-5) and annually (after year 5) is recommended for evaluation of overall survival and late toxicities (see section 7.7.)

[3] Interval of treatment administration, max. delay of therapy 4 weeks (for details see section 6.2). Additional gadolinium-enhanced brain MRI in case delay exceeds 2 weeks. Restart of treatment only in case of at least SD

[4] d-8 of HCT matches d22 of last cycle of MARTA. Max. delay of therapy 4 weeks (for details see section 6.2.1). Additional gadolinium-enhanced brain MRI in case delay exceeds 2 weeks. Restart of treatment only in case of at least SD

[5] Informed consent must be obtained before any study specific screening examination

[6] Maintenance treatment administration day 1-5 every 4 weeks for 6 months. Beginning within 5 days after RA II. Max. delay of therapy 4 weeks (for details see section 6.2). Unless clinically indicated otherwise, visits will be performed every 3 months during maintenance therapy.

[7] Hematology: white blood count (WBC), neutrophils, hemoglobin and platelets

[8] Blood chemistry: creatinine, total bilirubin, ALAT, ASAT, LDH, estimated GFR (MDRD), (and Gamma-GT only performed at screening); only LDH (at screening), estimated GFR (MDRD) and creatinine have to be documented in eCRF.

[9] Ultrasound to exclude third space fluid accumulation prior to MTX administration. If third space fluid accumulation has already been excluded during the screening period by means of (PET-) CT and clinically no new suspicious indications arise, a renewed evaluation before the start of the pre-phase therapy is not mandatory. This examination can also be omitted if the patient’s medical history and thorough physical examination do not suggest any third space fluid accumulation.

[10] If CT is suspicious at diagnosis: adequate further diagnostics by investigator’s decision, e.g. FDG-PET, biopsy. Note: FDG-PET-/CT can be performed at screening instead of CT neck to pelvis, refer to section 7.8.15.

[11] Only performed if positive at previous examination/clinically indicated; until results are negative or in case of participation in the translational program on condition that additional informed consent is available

[12] Only performed after excluding increased intracranial pressure by brain MRI; cytology, FACS and protein examination

[13] Beginning with RA II / premature EOT every 12 months during follow-up period

[14] During maintenance treatment in arm A, MRT assessment will be done every 3 months, unless it is clinically indicated otherwise

[15] If clinically indicated

[16] Central radiology assessment in the maintenance and/or follow-up period only if measureable lesions are still present and in case of recurrence. Shipment for central review as soon as possible after screening and after RA II (including assessments at RA I), after EOT (arm A) and in case of recurrence for the individual patient. In case of persisting measurable lesions, shipment during follow-up will be performed once at the end of the study for each trial site.

[17] Beginning with Follow-up MoCA every 12 months as screening test; subsequent neuropsychological battery only if 24-26 points in MoCA or if MoCA result deviates ≥4 points in comparison to previous test. If premature EOT occurs during maintenance treatment the neuropsychological battery does not have to be performed if it was done at RAII. (for details see 5.2.2)

[18] Subjective evaluation of trial participation questionnaire Q1 will be done after the patient was informed about the trial and has given informed consent, it may be done after start of treatment during the pre-phase treatment in-patient visit.

[19] Subjective evaluation of trial participation questionnaire Q2_Q3 will be done in arm A at RA II, EOT Visit (6 months after RA II) and premature EOT Visit. In arm B at RA II, premature EOT Visit and onetime during FU (6 months after RA II).

[20] Stem cell harvest is planned after the first cycle of R-MP in the control arm and after first cycle of R-MTX-AraC in the experimental arm. If stem cell harvest is unsuccessful at this time point further attempts can be made after cycle 2 and 3 in arm A or after cycle 2 in arm B before HCT.

[21] During screening period on condition that additional informed consent is available, additional biological specimen have to be taken before IMP administration (1 EDTA tube à 9 ml, 3 streck tubes à 10 mL, 1 Serum tube à 9 mL, 1 CSF à 4-5mL, tumor FFPE, bone marrow).

In arm **A** blood and CSF samples will be taken at randomization, RA II and EOT and in case of relapse and progressive disease. At premature end of treatment visit and during follow up period these samples will be taken only in case of relapse or progressive disease (1 EDTA à 9 mL, 3 streck tubes à 10 mL, 1 Serum tube à 9 mL, 1 CSF à 4-5mL).
In arm **B** blood and CSF samples will be taken at randomization, RAI and RAII (EOT) and in case of relapse and progressive disease. At premature end of treatment visit and during follow up period these samples will be taken only in case of relapse and progressive disease. (1 EDTA à 9 mL, 3 streck tubes à 10 mL, 1 Serum tube à 9 mL, 1 CSF à 4-5mL).
Respective collecting and shipping material will be provided by the University Hospital Freiburg. For details on translational project and sample handling and logistics see section 7.9 and study specific instruction sheet for shipping and sample handling respectively.

[22] Only performed if patient participates in the translational program (additional informed consent required); cytology, FACS and protein examination not mandatory.

[23] Only serious adverse events related to IMP as per investigator's judgement

[24] Where this is not considered standard evaluation for pulmonary function: Comparable pulmonary function tests can be used (refer to section 7.8.12).

[25] Shipping to central pathology should be arranged as soon as possible after registration in this trial.

[26] Patients eligible for HCT-ASCT defined by the EBL score (at most 1 of the 3 following conditions may apply: ECOG PS > 1, Barthel Index of ADL < 20 and Lachs geriatric screening > 3), improvement of PS after pre-phase treatment or clinical judgement by the treating physician after discussion with the study expert team. Discussion with the study expert team should take place from day 7-14 of prephase treatment in a weekly organised virtual meeting.

[27]If PET-CT was done at screening, bone marrow examination is not mandatory at screening.
